# Cryptosporidiosis in HIV-positive patients and related risk factors: A systematic review and meta-analysis

**DOI:** 10.1051/parasite/2020025

**Published:** 2020-04-30

**Authors:** Ehsan Ahmadpour, Hanie Safarpour, Lihua Xiao, Mehdi Zarean, Kareem Hatam-Nahavandi, Aleksandra Barac, Stephane Picot, Mohammad Taghi Rahimi, Salvatore Rubino, Mahmoud Mahami-Oskouei, Adel Spotin, Sanam Nami, Hossein Bannazadeh Baghi

**Affiliations:** 1 Research Center for Evidence Based Medicine (RCEBM), Tabriz University of Medical Sciences 5166-15731 Tabriz Iran; 2 Student Research Committee, Tabriz University of Medical Sciences 5166-15731 Tabriz Iran; 3 College of Veterinary Medicine, South China Agricultural University 510642 Guangzhou China; 4 Department of Parasitology and Mycology, Faculty of Medicine, Mashhad University of Medical Sciences 91779-48964 Mashhad Iran; 5 Iranshahr University of Medical Sciences 99147-86138 Iranshahr Iran; 6 Clinic for Infectious and Tropical Diseases, Clinical Centre of Serbia 11000 Belgrade Serbia; 7 University Lyon, ICBMS UMR 5246 CNRS-INSA-CPE & Institute of Parasitology and Medical Mycology, Croix-Rousse Hospital, Hospices Civils de Lyon 69004 Lyon France; 8 Center for Health Related Social and Behavioral Sciences Research, Shahroud University of Medical Sciences 36147-73947 Shahroud Iran; 9 Department of Biomedical Sciences, University of Sassari 07100 Sardinia Italy; 10 Department of Parasitology and Mycology, Faculty of Medicine, Tabriz University of Medical Sciences 5166-15731 Tabriz Iran; 11 Immunology Research Center, Tabriz University of Medical Sciences 5166-15731 Tabriz Iran; 12 Drug Applied Research Center, Tabriz University of Medical Sciences 5166-15731 Tabriz Iran; 13 Infectious and Tropical Diseases Research Center, Tabriz University of Medical Sciences 5166-15731 Tabriz Iran

**Keywords:** *Cryptosporidium* infection, HIV, AIDS, Systematic review

## Abstract

*Cryptosporidium* is one of the major causes of diarrhea in HIV-positive patients. The aim of this study is to systematically review and meta-analyze the prevalence of *Cryptosporidium* in these patients. PubMed, Science Direct, Google Scholar, Web of Science, Cochrane and Ovid databases were searched for relevant studies dating from the period of 1 January 2000 to 31 December 2017. Data extraction for the included studies was performed independently by two authors. The overall pooled prevalence was calculated and subgroup analysis was performed on diagnostic methods, geographical distribution and study population. Meta-regression was performed on the year of publication, proportion of patients with diarrhea, and proportion of patients with CD4 < 200 cells/mL. One hundred and sixty-one studies and 51,123 HIV-positive participants were included. The overall pooled prevalence of *Cryptosporidium* infection in HIV-positive patients was 11.2% (CI95%: 9.4%–13.0%). The pooled prevalence was estimated to be 10.0% (CI95%: 8.4%–11.8%) using staining methods, 13.5% (CI95%: 8.9%–19.8%) using molecular methods, and 26.3% (CI95%: 15.0%–42.0%) using antigen detection methods. The prevalence of *Cryptosporidium* in HIV patients was significantly associated with the country of study. Also, there were statistical differences between the diarrhea, CD4 < 200 cells/mL, and antiretroviral therapy risk factors with Cryptosporidiosis. Thus, *Cryptosporidium* is a common infection in HIV-positive patients, and safe water and hand-hygiene should be implemented to prevent cryptosporidiosis occurrence in these patients.

## Abstract (fr)

*Cryptosporidium* est l’une des principales causes de diarrhée chez les patients séropositifs pour le VIH. Le but de cette étude est de revoir et méta-analyser systématiquement la prévalence de *Cryptosporidium* chez ces patients. Les bases de données PubMed, Science Direct, Google Scholar, Web of Science, Cochrane et Ovid ont été recherchées pour des études pertinentes datant du 1er janvier 2000 au 31 décembre 2017. L’extraction des données pour les études incluses a été réalisée indépendamment par deux auteurs. La prévalence globale combinée a été calculée et une analyse en sous-groupes a été effectuée sur les méthodes de diagnostic, la répartition géographique et la population étudiée. Une méta-régression a été réalisée pour l’année de publication, la proportion de patients atteints de diarrhée et la proportion de patients avec CD4 < 200 cellules/mL. Cent soixante et une études et 51,123 participants séropositifs ont été inclus. La prévalence globale combinée de l’infection à *Cryptosporidium* chez les patients VIH-séropositifs était de 11,2 % (IC95 % : 9,4 %–13,0 %). La prévalence regroupée a été estimée à 10,0 % (IC95 % : 8,4 %–11,8 %) en utilisant des méthodes de coloration, 13,5 % (IC95 % : 8,9 %–19,8 %) en utilisant des méthodes moléculaires et 26,3 % (IC95 % : 15,0 %–42,0 %) en utilisant des méthodes de détection d’antigènes. La prévalence de *Cryptosporidium* chez les patients infectés par le VIH était significativement associée au pays d’étude. En outre, il existe des différences statistiques entre la diarrhée, les CD4 < 200 cellules/mL et les facteurs de risque du traitement antirétroviral avec la cryptosporidiose. Ainsi, *Cryptosporidium* est une infection courante chez les patients séropositifs, et une eau salubre et une hygiène des mains doivent être mises en œuvre pour prévenir la survenue de cryptosporidiose chez ces patients.

## Introduction

*Cryptosporidium* is an intracellular protozoan parasite that infects the gastrointestinal epithelium of a wide range of animals as well as humans, and causes diarrheal disease [29, 103]. Among the 38 species of *Cryptosporidium* currently recognized*, Cryptosporidium hominis* and *Cryptosporidium parvum* are responsible for the majority of human infections [43]. However, other species including *C. meleagridis, C. canis, C. felis*, and *C. muris* have been identified in immunocompromized patients [178]. Transmission of the infection is most common by the fecal-oral route, via the consumption of contaminated water and food, and contact with infected persons or animals [29]. Infection in immunocompetent patients is either asymptomatic or presents with profuse acute or persistent watery diarrhea, nausea and vomiting, stomach cramps, and occasionally fever that lasts approximately 2 weeks. However, in patients with immune deficiencies, the infection might cause prolonged symptoms and lead to chronic diarrhea that lasts more than 2 months, or fulminant diarrhea with more than 2 L of watery stools per day [29].

It is estimated that in 2016, 36.7 million people were infected with HIV worldwide. During the onset of the AIDS epidemic in the early 1980s *Cryptosporidium* became widely recognized as a human pathogen [160]. Diarrhea is a common problem in AIDS patients and about 30%–60% of patients in developed countries and 90% in developing countries experience diarrhea [44]. Diarrhea significantly influences quality of life and can lead to complications such as dehydration, malnutrition, weight loss and even death [101]. Cryptosporidiosis was considered one of the original AIDS-defining illnesses and a major risk factor for mortality compared to other AIDS-defining illnesses [32]. The prevalence of *Cryptosporidium* in immunocompetent patients varies widely, ranging from 0% to 10%, depending on country socioeconomic status [28]. Several studies have investigated the prevalence of *Cryptosporidium* in HIV-positive patients and have reported a wide range of estimates in different settings.

The aim of the study was to systematically review and meta-analyze the worldwide prevalence and geographic distribution of *Cryptosporidium* in HIV-positive patients and to compare the estimated prevalence using different diagnostic methods.

## Methods

### Search strategy and study selection

We performed this systematic review and meta-analysis according to the PRISMA (Preferred Reporting Items for Systematic Reviews and Meta-Analyses) statement [87]. PubMed, Science Direct, Google Scholar, Web of Science, Cochrane and Ovid databases were searched from 1 January 2000 to 31 December 2017 restricted to the English language using the following keywords: “*Cryptosporidium*”, “cryptosporidiosis”, “HIV”, “immunodeficiency”, “acquired immune deficiency syndrome”, or “AIDS”. After removing duplicate records, two authors independently reviewed the titles and/or abstracts of all records identified by the search. Full-texts were retrieved and evaluated for potentially relevant studies. All disagreements were resolved by consensus.

### Inclusion and exclusion criteria

Studies were included in the systematic review and meta-analysis if the study was performed on HIV/AIDS patients with or without diarrhea and the prevalence of *Cryptosporidium* was evaluated using staining, antigen detection or molecular methods. Conference abstracts, animal studies, case reports, comments, and reviews were excluded. When duplicate reports of the same research were suspected, the paper reporting more relevant data was included.

### Data extraction

Data extraction was performed independently by two authors and the following information was extracted: first author, year of publication, country of study, average level of income in the country of study, region of study, study design, number of HIV/AIDS participants, sex ratio of participants, mean age, diagnostic methods, number of participants co-infected with *Cryptosporidium*, number of participants with CD4 counts < 200 cell/mm^3^, and number of participants with diarrhea. The region of study was determined according to the WHO Global Burden of Disease Regions [176]. The level of income was retrieved from the 2017 World Bank classification of countries by income [175].

### Meta-analysis

Comprehensive meta-analysis 2.2 (Biostat Inc., USA) was used to calculate the pooled prevalence using a random-effects model. Heterogeneity was assessed using the *I*^2^ index and Cochran-Q test. An *I*^2^ index >70% or a significant Cochran-Q test indicated heterogeneity [37]. Also, publication bias was assessed using Egger’s intercept and visual inspection of the funnel plot. Univariate analysis was performed on the following risk factors and variables: diagnostic method, country of study, average level of income in the country of study, region of study, number of participants >100, proportion of patients with diarrhea, and proportion of patients with low CD4 counts. Meta-regression was performed using the method of moments on the following variables: year of publication, the proportion of patients with diarrhea, and proportion of patients with low CD4 counts.

In all analyses, if a study used multiple diagnostic methods, we preferred the prevalence estimated using molecular methods to the other two, and staining methods to antigen detection methods. This procedure was implemented for all analyses except in the subgroup analysis of diagnostic methods. In these studies, all estimates of prevalence using different diagnostic methods were included. Publication bias was assessed using Egger’s regression and visual inspection of the Funnel plot. A significant Egger’s regression and an asymmetric Funnel plot indicated publication bias [37]. The level of significance for all tests was *p* < 0.05.

## Results

### Search results

**Figure 1:**
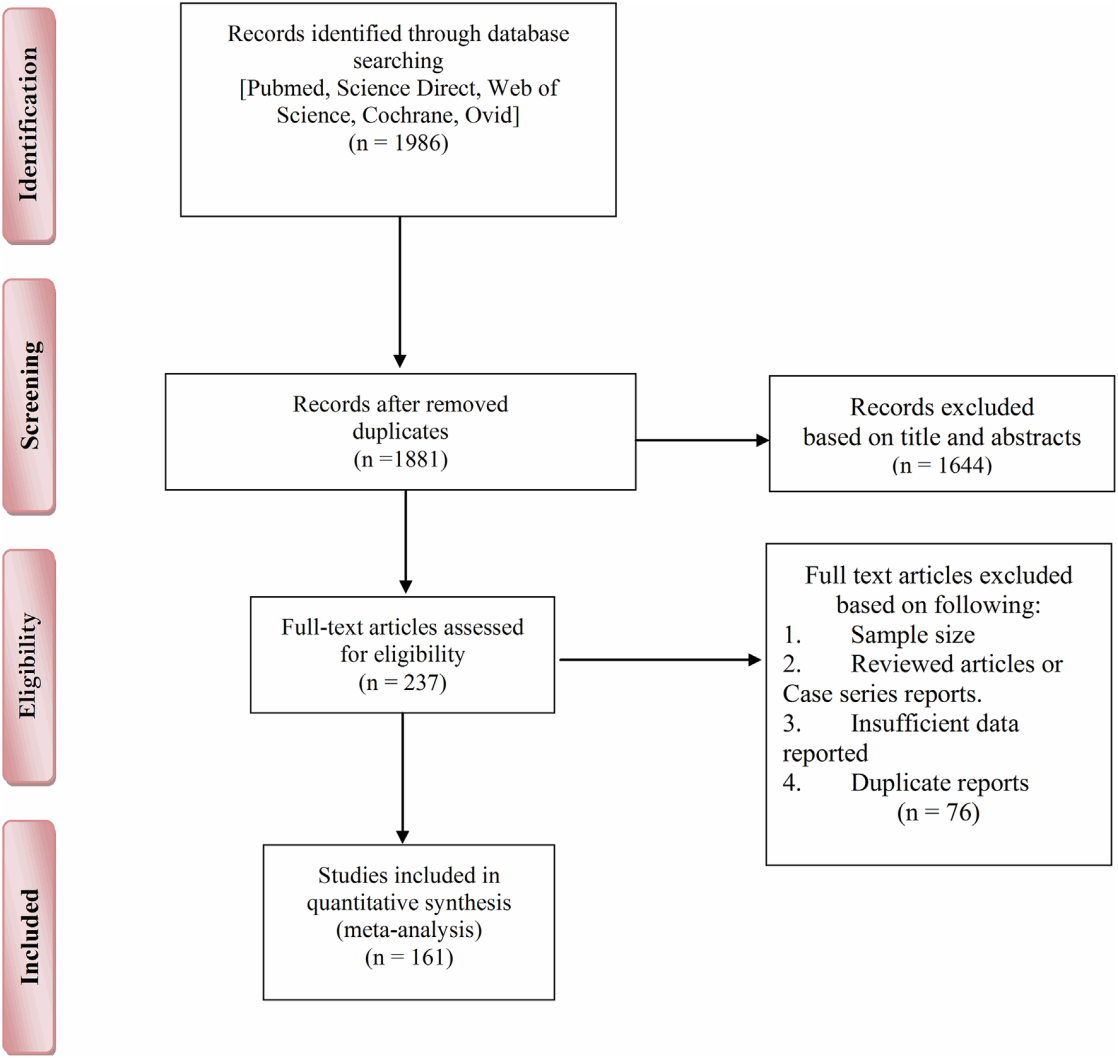
Flowchart describing the study design.

After removing duplicates, titles and/or abstracts of 1986 records retrieved by the search were screened and 237 studies were selected to be reviewed in more detail using their full-texts. Of these, 161 studies fit the inclusion criteria and were included in the systematic review and meta-analysis (Fig. 1).

### Characteristics of studies

A total of 51,123 HIV/AIDS patients participated in these studies of which 5408 patients were co-infected with *Cryptosporidium*. The overall male to female ratio was 61.2% to 38.8% (M:F = 1.58:1) among all participants and 67.2% to 32.3% (M:F = 2.08:1) among infected participants. The mean age of participants in the included studies was 33.9 years (ranged from 10 months to 45 years). In total, studies from 40 countries worldwide were included. The countries with the most included studies were India (25%, 41/161), Ethiopia (11%, 18/161), Brazil (8%, 12/161), Nigeria and Iran (6%, 10/161). More than 40% of studies were performed in lower middle-income countries (68/161), followed by upper-middle-income countries (32%, 52/161), low-income countries (20%, 33/161) and only 5% were performed in high-income countries (8/161). Studies were also categorized based on the WHO Global Burden of Disease Regions with 33% (53/161) of studies coming from the African region, 6% (10/161) from Eastern Mediterranean countries, 3% (5/161) from the European region, 14% (23/161) from the Americas, 34% (53/161) from the South-East Asian region, and 11% (17/161) from the Western Pacific region. In terms of study design, 80% (128/161) of studies were cross-sectional, 12% (20/161) were a cohort, 7% (12/161) were case-control, and one was a case-series. Staining, antigen detection, and molecular methods were used to diagnose *Cryptosporidium* infection in 87% (140/161), 12% (19/161), and 17% (28/161) of studies, respectively (Table 1). Some of the studies used several methods at the same time to confirm presence of *Cryptosporidium*.

**Table 1:** Baseline characteristics of the included studies.

Paper ID	First author	Year	Country/State	Number of participants	Number infected	Diagnostic method	Patients with diarrhea	Patients with CD4<200	Ref.
1	Inungu J	2000	Louisiana	6913	239	Staining	NR	NR	[62]
2	Chokephaibulkit K	2001	Thailand	82	7	Ziehl-Neelsen	100.00%	NR	[31]
3	Gassama A	2001	Senegal	318	15	Ziehl-Neelsen	49.70%	NR	[46]
4	Lebbad M	2001	Guinea-Bissau	37	9	Ziehl-Neelsen	NR	NR	[85]
5	Wiwanitkit V	2001	Thailand	60	2	Odine and Modified Trichromes	46.70%	41.70%	[174]
6	Brink AK	2002	Uganda	358	18	Ziehl-Neelsen	70.10%	NR	[22]
7	Joshi M	2002	India	94	8	Ziehl-Neelsen	NR	NR	[70]
8	Kumar SS	2002	India	150	14	Ziehl-Neelsen	66.70%	NR	[81]
9	Leav BA	2002	Congo	101	25	Ziehl-Neelsen	NR	NR	[84]
10	Mohandas K	2002	India	120	13	Ziehl-Neelsen	67.50%	NR	[99]
11	Saksirisampant W	2002	Thailand	156	20	Ziehl-Nelson	NR	NR	[129]
12	Wanachiwanawin D	2002	Thailand	95	3	Ziehl-Neelsen	100.00%	NR	[168]
13	Adjei A	2003	Ghana	21	6	Ziehl-Neelsen	100.00%	NR	[4]
14	Arenas-Pinto A	2003	Venezuela	304	45	Ziehl-Neelsen	71.40%	NR	[12]
15	Cama VA	2003	Peru	2672	354	Ziehl-Neelsen	NR	NR	[23]
16	Cranendonk R	2003	Malawi	348	16	Phenol-auramine-O-fluorescence	49.80%	NR	[33]
17	Shenoy S	2003	India	120	21	Ziehl-Neelsen	100.00%	NR	[138]
18	Silva CV	2003	Brazil	52	3	Safranin/Methylene Blue	NR	NR	[142]
19	Singh A	2003	India	100	47	Staining	NR	NR	[143]
20	Carcamo C	2004	Peru	294	39	Modified Safranin	50.00%	NR	[24]
21	Ribeiro PC	2004	Brazil	75	7	Safranin/Methylene Blue	NR	NR	[125]
22	Zali MR	2004	Iran	206	3	Ziehl-Neelsen	13.60%	NR	[183]
23	Certad G	2005	Venezuela	397	59	Ziehl-Neelsen	75.60%	NR	[26]
24	Guk SM	2005	Korea	67	7	Ziehl-Neelsen	NR	NR	[54]
25	Houpt ER	2005	Tanzania	127	22	IFA	48.00%	NR	[58]
26	Lim YA	2005	Malaysia	66	2	Ziehl-Neelsen	9.10%	NR	[89]
27	Marques FR	2005	Brazil	94	8	Ziehl-Neelsen, ELISA	NR	NR	[91]
28	Pinlaor S	2005	Thailand	78	9	Ziehl-Neelsen	32.10%	NR	[122]
29	Sadraei J	2005	India	200	84	Ziehl-Neelsen	38.00%	41.00%	[128]
30	Silva CV	2005	Brazil	100	4	Safranin/Methylene Blue, ELISA	38.00%	NR	[141]
31	Tadesse A	2005	Ethiopia	70	20	Ziehl-Neelsen	100.00%	NR	[148]
32	Tumwine JK	2005	Uganda	91	67	IFA	NR	NR	[158]
33	Adhikari NA	2006	Nepal	112	6	Ziehl-Neelsen	NR	NR	[3]
34	Chhin S	2006	Cambodia	80	36	Ziehl-Neelsen	50.00%	NR	[30]
35	Navarro-i-Martinez L	2006	Colombia	103	6	PCR, Ziehl-Neelsen	NR	NR	[102]
36	Oguntibeju OO	2006	Lesotho	60	6	Ziehl-Neelsen	56.70%	NR	[109]
37	Sarfati C	2006	Cameroon	154	6	Ziehl-Neelsen	28.60%	NR	[135]
38	de Oliveira-Silva MB	2007	Brazil	359	31	Ziehl-Neelsen	70.20%	NR	[36]
39	Dwivedi KK	2007	India	75	25	Ziehl-Neelsen	66.70%	NR	[40]
40	Hung CC	2007	Taiwan	332	4	PCR, Ziehl-Neelsen	NR	40.10%	[59]
41	Ramakrishnan K	2007	India	80	23	Ziehl-Neelsen	NR	NR	[124]
42	Rossit AR	2007	Brazil	55	34	ELISA	16.40%	NR	[127]
43	Stark D	2007	Australia	628	14	Modified iron-hematoxylin	100.00%	NR	[145]
44	Taherkhani H	2007	Iran	75	20	Ziehl-Neelsen	NR	NR	[149]
45	Vignesh R	2007	India	245	7	Ziehl-Neelsen	100.00%	NR	[164]
46	Bachur TP	2008	Brazil	582	47	Ziehl-Neelsen	NR	NR	[17]
47	Gupta S	2008	India	113	9	Ziehl-Neelsen	30.10%	NR	[56]
48	Jayalakshmi J	2008	India	89	11	Ziehl-Neelsen, ELISA	100.00%	NR	[68]
49	Kaushik K	2008	India	206	27	PCR, Ziehl-Neelsen, ELISA	48.10%	32.50%	[75]
50	Nuchjangreed C	2008	Thailand	46	2	PCR, Ziehl-Neelsen	28.30%	NR	[107]
51	Raccurt CP	2008	Haiti	74	45	PCR	NR	NR	[123]
52	Tuli L	2008	India	366	146	Ziehl-Neelsen	100.00%	64.50%	[156]
53	Werneck-Silva AL	2008	Brazil	690	1	Ziehl-Neelsen	NR	NR	[173]
54	Zaidah AR	2008	Malaysia	59	9	PCR, Ziehl-Neelsen	NR	NR	[182]
55	Zavvar M	2008	Iran	35	21	PCR, Ziehl-Neelsen	NR	NR	[184]
56	Assefa S	2009	Ethiopia	214	43	Ziehl-Neelsen	NR	NR	[14]
57	Daryani A	2009	Iran	64	6	Ziehl-Neelsen	NR	NR	[34]
58	Dillingham RA	2009	Haiti	243	39	Ziehl-Neelsen	NR	100.00%	[39]
59	Gautam H	2009	India	43	7	ELISA	NR	100.00%	[47]
60	Kulkarni SV	2009	India	137	16	Ziehl-Neelsen	NR	47.40%	[80]
61	Kurniawan A	2009	Indonesia	318	30	Ziehl-Neelsen	NR	NR	[82]
62	Lule JR	2009	Uganda	879	30	Ziehl-Neelsen	NR	29.90%	[90]
63	Saksirisampant W	2009	Thailand	90	31	PCR, Ziehl-Neelsen	78.90%	NR	[130]
64	Uppal B	2009	India	100	3	ELISA	50.00%	NR	[161]
65	Dehkordy AB	2010	Iran	33	3	ELISA	NR	NR	[38]
66	Getaneh A	2010	Ethiopia	192	48	Ziehl-Neelsen	NR	NR	[49]
67	Idris NS	2010	Indonesia	22	1	Ziehl-Neelsen	NR	NR	[61]
68	Kashyap B	2010	India	64	8	Safranin-methylene blue	NR	48.40%	[74]
69	Tuli L	2010	India	450	163	Ziehl-Neelsen	100.00%	NR	[157]
70	Akinbo FO	2011	Nigeria	2000	80	Ziehl-Neelsen	NR	12.80%	[8]
71	Alemu A	2011	Ethiopia	188	82	Ziehl-Neelsen	NR	NR	[10]
72	Cardoso LV	2011	Brazil	500	1	Ziehl-Neelsen	28.60%	NR	[25]
73	Erhabor O	2011	Nigeria	105	3	Ziehl-Neelsen	24.80%	NR	[41]
74	Kucerova Z	2011	Russia	46	19	ELISA	NR	NR	[79]
75	Lim YA	2011	Malaysia	122	27	Ziehl-Neelsen	NR	NR	[88]
76	Ojurongbe O	2011	Nigeria	96	52	Ziehl-Neelsen	NR	NR	[112]
77	Patel SD	2011	India	100	20	Ziehl-Neelsen	32.00%	NR	[118]
78	Santos RB	2011	Brazil	1010	4	Staining	NR	NR	[134]
79	Srisuphanunt M	2011	Thailand	152	33	PCR, Ziehl-Neelsen, ELISA	NR	NR	[144]
80	Stensvold CR	2011	Denmark	96	1	Staining	NR	13.50%	[146]
81	Boaitey YA	2012	Ghana	500	70	Ziehl-Neelsen	51.60%	NR	[21]
82	Iqbal A	2012	Malaysia	346	18	PCR	NR	NR	[63]
83	Izadi M	2012	Iran	47	7	Ziehl-Neelsen	NR	NR	[65]
84	Jha AK	2012	India	154	87	Ziehl-Neelsen	NR	35.10%	[69]
85	Kange’the E	2012	Kenya	155	7	Ziehl-Neelsen	NR	NR	[72]
86	Khurana S	2012	India	671	40	PCR, Ziehl-Neelsen, ELISA	NR	NR	[77]
87	Lehman LG	2012	Cameroon	201	13	Ziehl-Neelsen	18.40%	NR	[86]
88	Masarat S	2012	India	45	45	Ziehl-Neelsen, ELISA	NR	NR	[92]
89	Netor Velasquez J	2012	Argentina	11	3	PCR	NR	NR	[105]
90	Ojuromi OT	2012	Nigeria	193	44	Ziehl-Neelsen	34.70%	NR	[111]
91	Pavie J	2012	France	143	8	Ziehl-Neelsen	59.40%	100.00%	[119]
92	Roka M	2012	Guinea	260	24	Ziehl-Neelsen	NR	NR	[126]
93	Sharma P	2012	India	970	44	Ziehl-Neelsen	NR	NR	[137]
94	Tian LG	2012	China	302	25	Ziehl-Neelsen	NR	NR	[153]
95	Vyas N	2012	India	366	75	Ziehl-Neelsen	72.70%	NR	[166]
96	Wang L	2013	China	683	10	PCR	44.50%	NR	[169]
97	Adamu H	2013	Ethiopia	378	32	Ziehl-Neelsen	45.30%	NR	[2]
98	Agholi M	2013	Iran	356	34	Ziehl-Neelsen	28.90%	52.80%	[5]
99	Ahmed NH	2013	India	242	40	Ziehl-Neelsen	NR	NR	[6]
100	Akinbo FO	2013	Nigeria	285	4	PCR	37.90%	15.80%	[9]
101	Assis DC	2013	Brazil	59	6	Ziehl-Neelsen	39.00%	NR	[15]
102	Ayinmode AB	2013	Nigeria	132	8	PCR	59.80%	13.60%	[16]
103	Bartelt LA	2013	South Africa	193	146	ELISA	NR	NR	[18]
104	Dash M	2013	India	115	14	Ziehl-Neelsen	NR	36.50%	[35]
105	Gupta K	2013	India	100	4	Ziehl-Neelsen	19.00%	32.00%	[55]
106	Janagond AB	2013	India	100	2	Ziehl-Neelsen	68.00%	30.00%	[67]
107	Rashmi KS	2013	India	90	15	Ziehl-Neelsen	NR	NR	[71]
108	Mathur MK	2013	India	544	135	Ziehl-Neelsen	73.50%	NR	[93]
109	Mehta KD	2013	India	100	2	Ziehl-Neelsen	NR	24.00%	[94]
110	Missaye A	2013	Ethiopia	272	2	Ziehl-Neelsen	NR	10.70%	[96]
111	Mohanty I	2013	India	250	13	Ziehl-Neelsen	80.00%	NR	[100]
112	Teklemariam Z	2013	Ethiopia	371	8	Ziehl-Neelsen	20.20%	27.00%	[151]
113	Tian LG	2013	China	79	8	Ziehl-Neelsen	NR	100.00%	[154]
114	Tiwari BR	2013	Nepal	745	23	Ziehl-Neelsen	33.30%	43.90%	[155]
115	Vyas N	2013	India	75	11	Ziehl-Neelsen	NR	42.70%	[167]
116	Zeynudin A	2013	Ethiopia	91	8	Ziehl-Neelsen	NR	NR	[185]
117	Adamu H	2014	Ethiopia	520	140	PCR	NR	NR	[1]
118	Blanco MA	2014	Guinea	171	31	PCR	NR	NR	[20]
119	Girma M	2014	Ethiopia	268	92	Ziehl-Neelsen	90.30%	69.80%	[52]
120	Omoruyi BE	2014	South Africa	35	23	PCR, Ziehl-Neelsen, ELISA	NR	NR	[113]
121	Paboriboune P	2014	Laos	137	9	Ziehl-Neelsen	43.10%	100.00%	[115]
122	Parghi E	2014	India	93	16	Ziehl-Neelsen	NR	19.40%	[117]
123	Samie A	2014	South Africa	106	30	PCR, Ziehl-Neelsen	NR	NR	[132]
124	Shimelis T	2014	Ethiopia	250	32	Ziehl-Neelsen	NR	NR	[139]
125	Taye B	2014	Ethiopia	316	3	Ziehl-Neelsen	NR	NR	[150]
126	Uppal B	2014	India	58	45	PCR, Ziehl-Neelsen, ELISA	NR	100.00%	[162]
127	Vouking MZ	2014	Cameroon	207	15	Ziehl-Neelsen	NR	NR	[165]
128	Wanyiri JW	2014	Kenya	164	56	PCR, Ziehl-Neelsen	42.70%	NR	[171]
129	Ahmed NH	2015	India	142	6	Ziehl-Neelsen	NR	NR	[7]
130	Angal L	2015	Malaysia	131	5	Ziehl-Neelsen	NR	18.30%	[11]
131	Asma I	2015	Malaysia	346	43	Ziehl-Neelsen	NR	NR	[13]
132	Fregonesi BM	2015	Brazil	17	4	Ziehl-Neelsen	NR	NR	[45]
133	Khalil S	2015	India	200	15	Ziehl-Neelsen	50.00%	50.00%	[76]
134	Kiros H	2015	Ethiopia	399	23	Ziehl-Neelsen	NR	16.80%	[78]
135	Mengist HM	2015	Ethiopia	180	7	Ziehl-Neelsen	NR	NR	[95]
136	Ojuromi OT	2015	Nigeria	90	4	PCR	74.40%	NR	[110]
137	Oyedeji OA	2015	Nigeria	52	10	Ziehl-Neelsen	NR	NR	[114]
138	Pavlinac PB	2015	Kenya	56	1	Ziehl-Neelsen	NR	NR	[120]
139	Petrincová A	2015	Slovak Republic	20	0	PCR	NR	NR	[121]
140	Tellevik MG	2015	Tanzania	33	8	PCR	NR	NR	[152]
141	Wumba RD	2015	Congo	242	13	PCR, Ziehl-Neelsen	34.30%	NR	[177]
142	Zhang L	2015	China	190	26	ELISA	NR	33.70%	[186]
143	Gholami R	2016	Iran	53	4	Ziehl-Neelsen	100.00%	100.00%	[51]
144	Hailu AW	2016	Ethiopia	81	6	Ziehl-Neelsen	NR	NR	[57]
145	Kaniyarakkal V	2016	India	200	2	Ziehl-Neelsen, Elisa	45.50%	100.00%	[73]
146	Kwakye-Nuako G	2016	Ghana	50	6	Ziehl-Neelsen	NR	46.00%	[83]
147	Mitra S	2016	India	194	57	Ziehl-Neelsen	NR	NR	[97]
148	Nsagha DS	2016	Cameroon	300	132	Ziehl-Neelsen	39.30%	25.30%	[106]
149	Salehi Sangani G	2016	Iran	80	1	Ziehl-Neelsen	NR	100.00%	[131]
150	Shah S	2016	India	45	6	Ziehl-Neelsen	60.00%	100.00%	[136]
151	Shimelis T	2016	Ethiopia	491	65	Ziehl-Neelsen	43.80%	56.20%	[140]
152	Eshetu T	2017	Ethiopia	223	7	Ziehl-Neelsen	NR	NR	[42]
153	Gedle D	2017	Ethiopia	323	19	Ziehl-Neelsen	NR	NR	[48]
154	Ghafari R	2017	Iran	250	27	PCR, Ziehl-Neelsen	NR	NR	[50]
155	Irisarri-Gutierrez MJ	2017	Mozambique	70	4	Ziehl-Neelsen	NR	NR	[64]
156	Obateru O.A	2017	Nigeria	238	131	Ziehl-Neelsen	NR	NR	[108]
157	Swathirajan CR	2017	India	829	19	Modified acid-fast	100.00%	NR	[147]
158	Ukwah BN	2017	Nigeria	251	17	PCR	100.00%	28.70%	[159]
159	Uysal HK	2017	Turkey	115	3	PCR, Ziehl-Neelsen	NR	NR	[163]
160	Yang Y	2017	China	46	2	Modified acid-fast	NR	NR	[180]
161	Yang Y	2017	China	14	3	Modified acid-fast	NR	NR	[181]

Abbreviations: ELISA: Enzyme-Linked Immunosorbent Assay, IFA: Immunofluorescence Assay, PCR: Polymerase Chain Reaction, NR: not reported.

### Statistical analysis

**Figure 2:**
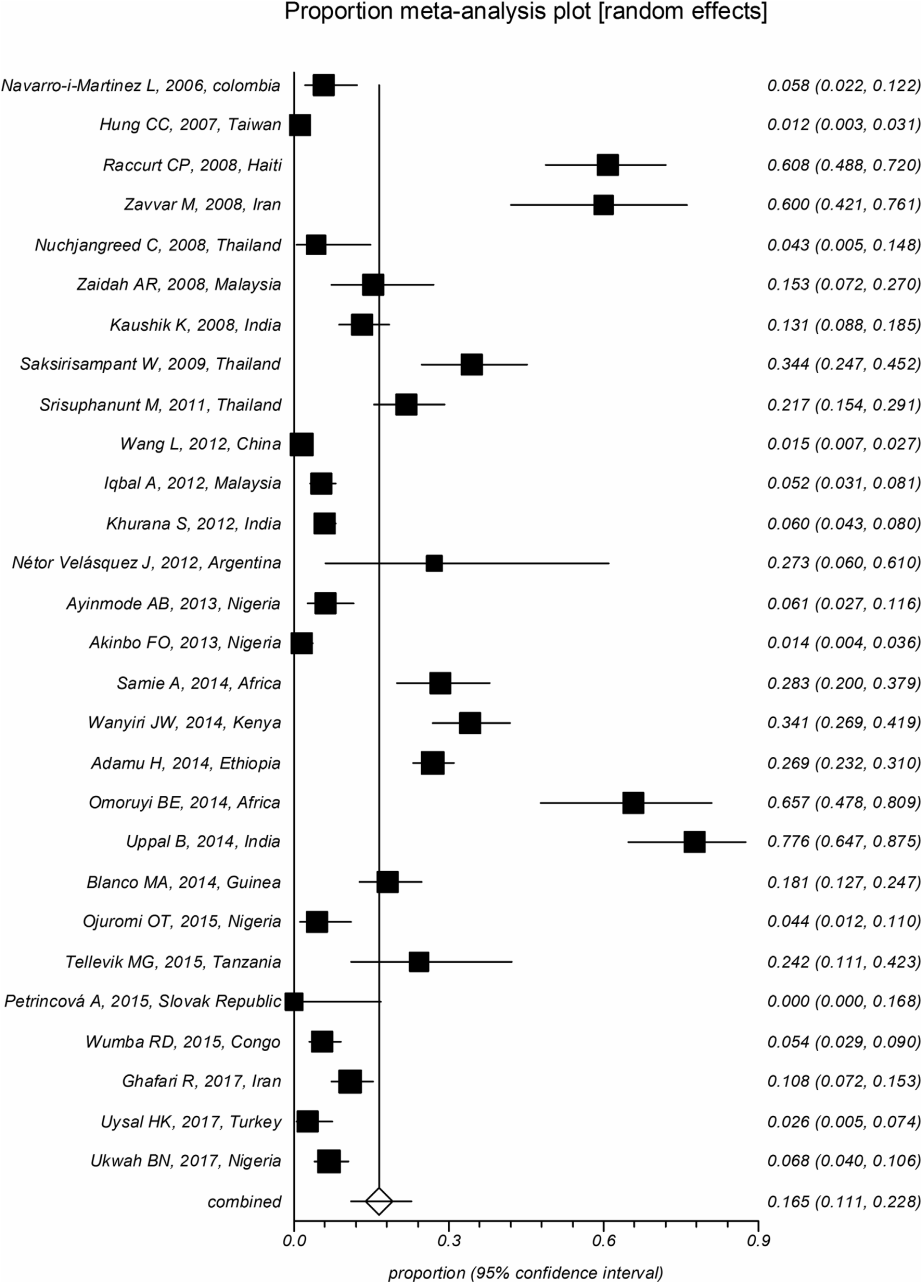
Forest plot diagram: The estimated pooled prevalence of *Cryptosporidium* infection in people with HIV infection by random-effect meta-analysis in included studies based on the PCR technique (first author, year of publication, and country). Note: The area of each square is proportional to the study’s weight in the meta-analysis, and each line represents the confidence interval around the estimate. The diamond represents the pooled estimate.

**Figure 3:**
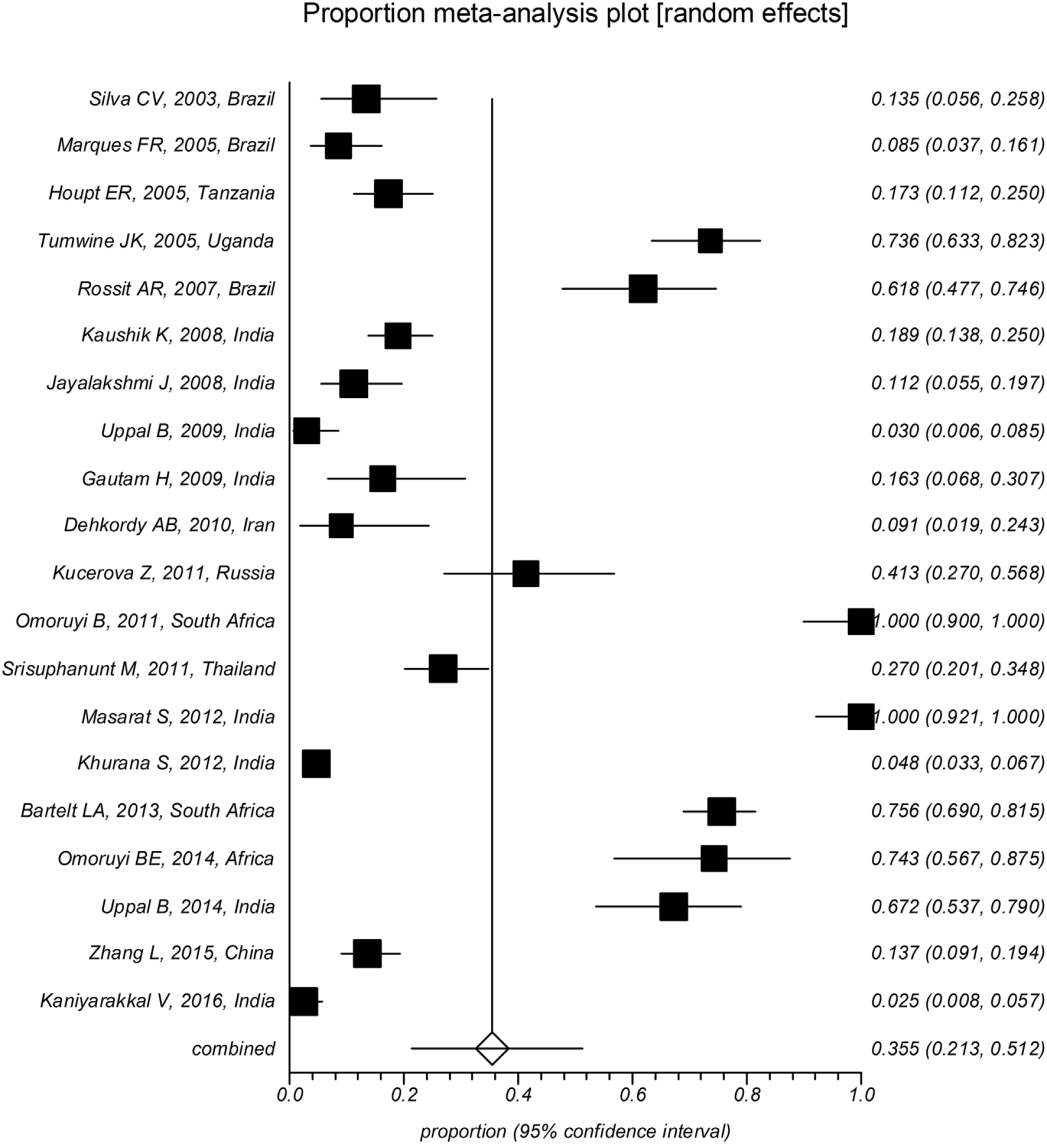
Forest plot diagram: The estimated pooled prevalence of *Cryptosporidium* infection in people with HIV infection by random-effect meta-analysis in included studies based on serological methods (first author, year of publication, and country). Note: The area of each square is proportional to the study’s weight in the meta-analysis, and each line represents the confidence interval around the estimate. The diamond represents the pooled estimate.

**Figure 4:**
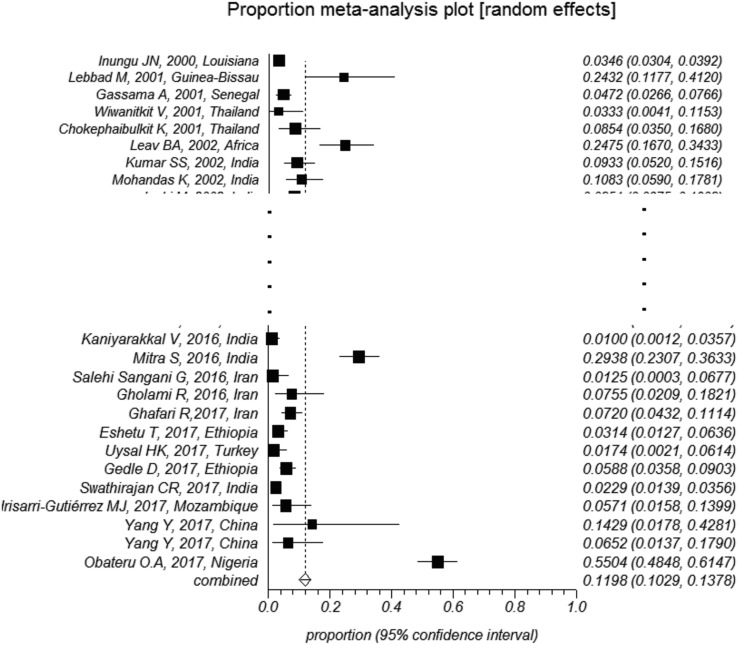
Forest plot diagram: The estimated pooled prevalence of *Cryptosporidium* infection in people with HIV infection by random-effect meta-analysis in included studies based on the staining method (first author, year of publication, and country). Note: The area of each square is proportional to the study’s weight in the meta-analysis, and each line represents the confidence interval around the estimate. The diamond represents the pooled estimate.

**Figure 5:**
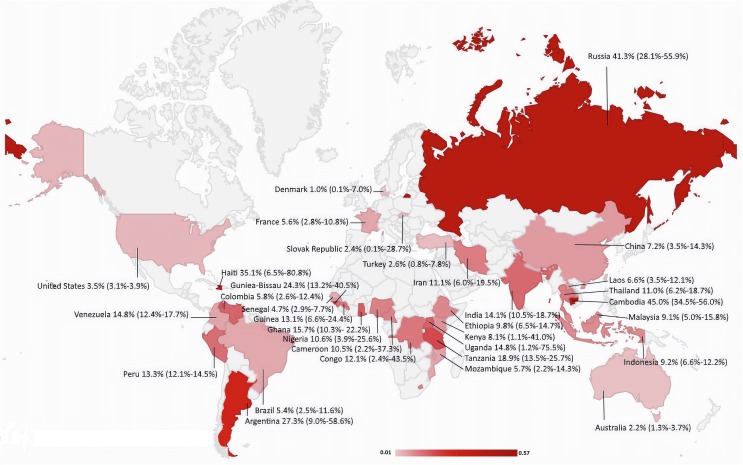
Pooled prevalence of *Cryptosporidium* in HIV-positive patients in different countries (source of image: https://commons.wikimedia.org/wiki/File:BlankMap-World.svg).

The overall pooled prevalence of *Cryptosporidium* infection in HIV-positive patients was 14.42% (CI95%: 12.61%–16.32%). Substantial heterogeneity with an *I*^2^ of 96.4% and a significant Cochran-Q test was observed. Different diagnostic methods were utilized to detect *Cryptosporidium* infection which significantly influenced the estimated prevalence (*p* < 0.05). The pooled prevalence was estimated to be 11.9% (CI95%: 10.2%–13.7%) using staining methods, 16.5% (CI95%: 11.1%–22.8%) using molecular methods, and 35.5% (CI95%: 21.3%–51.2%) using antigen detection methods (Figs. 2–4). The country of studies significantly affected the estimated pooled prevalence (*p* < 0.05). South Africa had the highest prevalence (57.0%, CI95%: 24.4%–84.5%), while Denmark had the lowest prevalence (1.0%, CI95%: 0.1%–7.0%), although very few studies were performed in these countries. Among countries where more than ten studies were included, India had the highest prevalence (14.1%, CI95%: 10.5%–18.7%), while Brazil had the lowest prevalence (5.4%, CI95%: 2.5%–11.6%). The geographical distribution of *Cryptosporidium* and HIV co-infection is shown in Figure 5.

**Figure 6:**
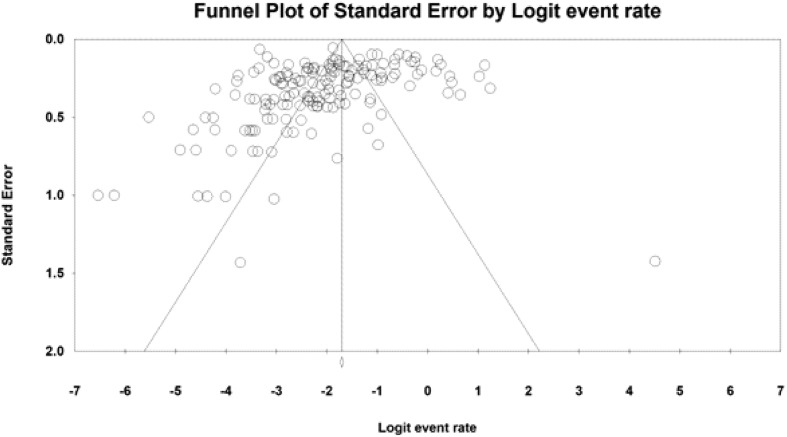
Funnel plot of standard error by logit event rate to assess publication or other types of bias across prevalence studies.

The prevalence in high-income countries was 4.1% (2.4%–6.9%), which was significantly lower than in countries with lower income (*p* < 0.05). However, no significant difference was observed between upper-middle, lower-middle and low-income countries (*p* = 0.43). Additionally, the prevalence was not significantly different across WHO Global Burden of Disease Regions (*p* = 0.46). The South-East Asia region, with a pooled estimate of 12.7% (CI95%: 9.7%–16.4%), had the highest prevalence. Studies including less than 100 participants reported a significantly higher prevalence (15.4%, CI95%: 11.8%–19.8%) compared to the studies with more than 100 participants (8.9%, CI95%: 7.2%–11.0%). The proportion of participants with diarrhea was reported in 42% (69/161) of studies. Additionally, meta-regression showed there is no statistically significant difference within prevalence rate, depending on the year of publication (*β* intercept = −0.013, *p* = 0.50). All subgroup meta-analyses were significantly heterogeneous (Table 2). Among these studies, meta-analysis showed that the proportion of participants with diarrhea and CD4 counts < 200 cells/mL significantly correlated with the pooled prevalence (*p* < 0.0001). Similarly, the proportion of participants who received ART significantly correlated with the pooled prevalence (*p* < 0.0001) (Table 3). Our study indicated that having diarrhea and having less than 200 CD4 cells μL, in HIV-infected patients, increase the risk of infection by Cryptosporidium, whereas using antiretroviral therapy in HIV-infected patients meaningfully decreases the risk of cryptosporidiosis. The funnel plot showing an asymmetric plot with studies missing on the right side and a statistically significant Egger’s regression suggest the possibility of publication bias (Fig. 6).

**Table 2:** Pooled prevalence of *Cryptosporidium* in HIV-positive patients and subgroup analyses.

Group	Number of studies	Pooled prevalence (CI 95%)	Heterogeneity		*p*-value
			*p* value	*I*^2^ (%)	
Diagnostic method					*p* < 0.05
Staining	140	10.0% (8.4%–11.8%)	<0.001	96.00	
Antigen detection	19	26.3% (15.0%–42.0%)	<0.001	96.90	
Molecular	28	13.5% (8.9%–19.8%)	<0.001	95.60	
Country*					*p* < 0.05
Brazil	12	5.4% (2.5%–11.6%)	<0.001	93.90	
China	6	7.2% (3.5%–14.3%)	<0.001	87.50	
Ethiopia	18	9.8% (6.5%–14.7%)	<0.001	95.70	
India	41	14.1% (10.5%–18.7%)	<0.001	95.90	
Iran	10	11.1% (6.0%–19.5%)	<0.001	89.40	
Malaysia	7	9.1% (5.0%–15.8%)	<0.001	86.60	
Nigeria	11	10.6% (3.9%–25.6%)	<0.001	98.30	
Thailand	8	11.0% (6.2%–18.7%)	<0.001	85.40	
Region					*p* = 0.46
African Region	53	11.9% (8.8%–16.0%)	<0.001	97.00	
Eastern Mediterranean Region	10	11.1% (6.0%–19.5%)	<0.001	89.40	
European Region	5	5.4% (1.0%–23.7%)	<0.001	92.00	
Region of the Americas	23	9.8% (6.4%–14.8%)	<0.001	97.30	
South-East Asia Region	53	12.7% (9.7%–16.4%)	<0.001	95.50	
Western Pacific Region	17	7.7% (4.7%–12.3%)	<0.001	92.60	
Income Level					*p* = 0.43
High income	8	4.1% (2.4%–6.9%)	<0.001	77.80	
Upper-middle income	52	10.4% (8.0%–13.5%)	<0.001	94.10	
Lower-middle income	68	13.1% (10.2%–16.6%)	<0.001	96.30	
Low income	33	10.9% (7.6%–15.2%)	<0.001	96.30	
Number of Participants					*p* < 0.05
<100	66	15.4% (11.8%–19.8%)	<0.001	91.00	
>100	95	8.9% (7.2%–11.0%)	<0.001	97.30	

* Only countries with more than 5 included studies are shown.

**Table 3:** Risk factors associated to *Cryptosporidium* infection in HIV patients.

Risk factors	No. of studies	Categories	OR (95% CI)	*I*² (inconsistency) %	Cochran Q	*p*-value
Sex	20	Male	1.11 (0.92–1.33)	0	18.96	*p* = 0.18
		Female				
Diarrhea	44	Yes	3.05 (2.23–4.18)	59.2	105.34	*p* < 0.0001
		No				
Antiretroviral therapy (ART)	19	Yes	2.02 (1.19–3.41)	65.3	51.85	*p* < 0.0001
		No				
CD4+	26	< 200 cells/ml3	5.84 (3.1–10.99)	88	207.75	*p* < 0.0001
		> 200 cells/ml3				
Water	3	Boiled	0.88 (0.51–1.50)	0	1.25	*p* = 0.34
		Tap				

## Discussion

Diarrhea caused by opportunistic intestinal protozoa is a common problem in HIV-infected patients. With a total number of 36 million HIV-infected patients and 11.2% prevalence of *Cryptosporidium* co-infection with HIV, approximately 4 million HIV patients are estimated to be infected with *Cryptosporidium* worldwide. The present meta-analysis of 161 studies published from 2000 to 2017 on the topic of *Cryptosporidium* infections in patients with HIV shows that the pooled worldwide prevalence of *Cryptosporidium* in patients with HIV is 14.4%. A systematic review previously assessed the worldwide prevalence of *Cryptosporidium* among patients with HIV, but did not establish the risk factors [170]. The prevalence of *Cryptosporidium* in the immunocompetent population has been estimated to be not more than 1% in high-income and 5%–10% in low-income countries [28]. In a case-control study, it was shown that HIV-positive patients had a 20-fold risk of becoming infected with *Cryptosporidium* [97, 98]. Therefore, in addition to a greater risk of developing symptomatic disease and having more severe and prolonged symptoms, patients with HIV have a greater risk of infection with *Cryptosporidium* [60].

Several mechanisms have been suggested to explain the susceptibility of AIDS patients to cryptosporidiosis. CD4 cells play a major role in the immune response to gastrointestinal pathogens, and it has been shown that low CD4 counts are associated with increased risk of infection with enteric parasites and chronic diarrhea [104]. Due to immunosuppression, symptoms of cryptosporidiosis in patients with AIDS are expressed differently in terms of severity, duration, and responses to drug treatment. It has been shown that there is a significant relationship between increased mortality rates and cryptosporidiosis in AIDS patients [19, 179]. Similarly, in the present meta-analysis, we showed that the patients with low CD4 counts had a higher prevalence rate of *Cryptosporidium* infection (*p* < 0.0001). It seems that IFN-*γ* is associated with T-cell memory and is a critical regulator of both innate and adaptive immune responses against *Cryptosporidium* infection. Also, the findings of immunological research suggest that *Cryptosporidium* induced an inflammatory response in intestinal epithelial cells. Accordingly, the higher expression of inflammatory and pro-inflammatory cytokines, such as CXCL-10 and substance P is present in AIDS patients (compared to AIDS patients without cryptosporidiosis or negative controls) [116]. The opportunistic parasites *Cryptosporidium* spp. are not only associated with the immune state in HIV-infected patients, but are also more evident with antiretroviral therapy. Utilization of chemoprophylaxis could increase the immunity of HIV-positive individuals and reduce the infection. Our findings suggested that in HIV-infected patients, especially with low CD4 counts, ART should be prescribed.

Substantial heterogeneity was observed between the studies included in this meta-analysis. In addition to using the random effects model, which incorporates some of this heterogeneity, we investigated possible causes of heterogeneity and compared the estimated prevalence in different subgroups and settings [37]. The diagnostic method that was used to detect *Cryptosporidium* infection significantly influenced the estimated prevalence. The included studies had utilized three main categories of diagnostic methods. PCR is considered the gold standard in diagnosing *Cryptosporidium* infection with an excellent sensitivity of 97% and specificity of 100%, but is not commonly used due to its high cost and high expertise requirement, especially in low-income countries [28]. The estimated pooled prevalence using PCR was 16.5%, which could be considered as the “real” prevalence. Conventional microscopy, most commonly using Ziehl-Neelsen staining, is an inexpensive and widely available method but has a low sensitivity of 75% [27]. The estimated pooled prevalence using staining methods was 11.9%, which was the lowest estimate among used diagnostic methods. Enzyme Immunoassays (EIA), based on detection of *Cryptosporidium* antigens, cost more than the staining methods and have a moderate to high diagnostic accuracy, with a sensitivity of 75%–93%. However, confirmatory testing has been suggested when using EIA, since some false-positive reactions have been confirmed [27, 28, 172]. The pooled prevalence using antigen detection methods was the highest among diagnostic methods with an estimate of 35.5%. In addition to false-positive reactions, we propose that the higher prevalence in studies that utilized EIA methods could be due to possible continued shedding of *Cryptosporidium* antigens in the stools, even after the resolution of infection, although this effect has not been studied.

The geographical distribution was another confounding factor. The estimated prevalence within countries was in a range of 1% in Denmark to 57% in South Africa. Among the countries with more than ten included studies, India (14.1%), Iran (11.1%) and Nigeria (10.6%) had the highest prevalence. The economic status of different countries could be the most probable explanation for these findings. The prevalence in high-income countries, with an estimate of 4.1%, was significantly lower than in middle and low-income countries, but there was no statistically significant difference between the estimated prevalence in the middle-income and low-income countries. Additionally, the source of drinking water can contribute to the different prevalence observed within different countries. A meta-analysis showed that drinking unsafe water significantly increases the risk of *Cryptosporidium* infection [53]. However, we were unable to evaluate its effect on prevalence since very few studies reported the sources of drinking water. Our study showed that the pooled prevalence across WHO Global Burden of Disease regions was not significantly different.

The association of *Cryptosporidium* prevalence and the proportion of symptomatic HIV patients has been investigated. No statistically significant difference was observed between the prevalence in studies with a high proportion of symptomatic patients and studies with a low proportion of symptomatic patients, although the meta-regression showed a correlation between prevalence and the proportion of symptomatic patients. Another significant confounding variable was the number of participants in the included studies. Studies with a lower number of participants reported higher prevalence rates. This could be due to the fact that lower sample sizes are associated with higher sampling error [133].

The studies also differed in the period when they were conducted, but meta-regression showed that the year of publication did not correlate to estimated prevalence. A meta-analysis suggested seasonality in the prevalence of *Cryptosporidium*, and showed that precipitation and temperature are strongly associated with the rate of infection [66]. However, it was not possible to investigate the impact of seasons and different climates on the prevalence in the present meta-analysis, due to the limited data reported. Nonetheless, the heterogeneity after considering these confounding variables was still high. Other unknown and uninvestigated differences in study design and population might exist, but it is not uncommon for meta-analyses to have high heterogeneity. In addition to high heterogeneity, our study was also limited by the publication bias. This occurs when the results of studies influence the decision of the author or publisher. Therefore, we recommend developing a database of HIV patients infected with *Cryptosporidium* to estimate the overall prevalence of cryptosporidiosis and the geographical and time distribution of infection more accurately.

## Conclusion

The prolonged and severe diarrhea caused by *Cryptosporidium* is associated with significant morbidity and mortality, especially in the HIV-infected population. This highlights the importance of preventive measures such as drinking safe water, using community-based or household water treatment systems, and education on hand hygiene after using toilets and before preparing food. Additionally, clinicians should consider early symptoms of cryptosporidiosis, such as diarrhea, in HIV patients, with the aim of initiating treatment early in the disease course. Also, patients with a CD4 count below 200 should receive prophylactic antiparasite treatment. If implemented correctly, these measures could lead to decreased morbidity, mortality, and transmission.
